# Menopausal characteristics and hormone replacement therapy in relation to long-term risk of cholecystectomy in women

**DOI:** 10.3389/fmed.2024.1446271

**Published:** 2024-11-27

**Authors:** Guan-Jun Ding, Wei Jiang, Jie-Qiong Lyu, Jie Ma, Guo-Chong Chen, Fu-Rong Li, Si-Jia Yang, Meng-Yuan Miao, Yong-Fei Hua

**Affiliations:** ^1^The Second Department of General Surgery, Ningbo No. 9 Hospital, Ningbo, China; ^2^Department of Hepatopancreatobiliary Surgery, Ningbo Medical Center Lihuili Hospital, Ningbo University, Ningbo, China; ^3^Department of Nutrition and Food Hygiene, School of Public Health, Suzhou Medical College of Soochow University, Suzhou, China; ^4^School of Public Health and Emergency Management, Southern University of Science and Technology, Shenzhen, China; ^5^Ningbo Municipal Center for Disease Control and Prevention, Ningbo, China

**Keywords:** cholecystectomy, gallbladder disease, hormone replacement therapy, menopausal characteristics, cohort study

## Abstract

**Background:**

Women are known to be at higher risk for gallbladder disease than men, suggesting a role of female hormones in the pathogenesis of gallbladder disease. This study aimed to assess menopausal characteristics, hormone replacement therapy (HRT) and their joint effect on long-term risk of cholecystectomy in women.

**Methods:**

A total of 184,677 women were included from the UK Biobank. Multivariable Cox regression models were used to estimate the associations of menopausal characteristics and HRT with risk of cholecystectomy. The joint influence of HRT use and the status and type of menopause on incident cholecystectomy were further evaluated.

**Results:**

During a median of 12.7 years of follow-up, 4,991 incident cases of cholecystectomy were identified. Natural menopause, regardless of menopausal age, was not associated with risk of cholecystectomy, while surgical menopause at a young age was associated with a higher risk of cholecystectomy. Ever use of HRT was associated with a higher risk of cholecystectomy. In particular, women who were surgically-menopausal and started HRT before menopause had the highest risk for cholecystectomy (hazard ratio = 2.28; 95% confidence interval: 1.70–3.04), when compared with women who were naturally-menopausal and never used HRT.

**Conclusions:**

Early surgical menopause and ever use of HRT was independently associated with the risk of cholecystectomy.

## Introduction

Gallbladder disease occurs largely as a result of formation of cholesterol gallstones, with the prevalence reaching 10%−30% in European countries (1). While most gallstones are clinically silent, up to 20% individuals with symptomatic gallstones have a 1%-2% likelihood annually of developing severe complications and requiring surgical removal of the gallbladder (2, 3). Cholecystectomy has greatly increased the number of hospitalizations and costs of healthcare resources, resulting in a substantial financial burden (4).

Women have a markedly higher risk of gallstones or cholecystectomy than men (5), indicating a role of sex steroid hormones in the pathogenesis of gallbladder disease. Estrogen has been suggested to have priming effects on the inflammatory and nociceptive pathways (6, 7), leading to symptoms that mimic cholecystitis. Elevated estrogen levels have been proposed to lead to supersaturation of biliary cholesterol, promoting the precipitation of solid cholesterol monohydrate crystals, which contributes to gallstone formation (8). However, evidence from epidemiological studies have been inconsistent regarding the relationship between length of women's reproductive years and gallbladder disease (9–11). On the other hand, several prospective observational studies have shown an increase in the risk of gallbladder disease or cholecystectomy associated with postmenopausal hormone replacement therapy (HRT) in women (12–15), supporting the lithogenicity of exogenous estrogen. As HRT use is commonly accompanied by early menopause (especially early surgical menopause), their independent and joint influences on the long-term risk of cholecystectomy remain to be assessed.

In the present study, using data from a large population-based prospective study (UK Biobank), we investigated the relationships of menopausal characteristics and HRT use as well as their joint influence on the risk of cholecystectomy in women.

## Methods

### Study population

The UK Biobank is a large prospective cohort study established to provide a resource for investigation of the genetic, environmental, and lifestyle factors associated with a wide range of diseases (16). From 2006 to 2010, the UK Biobank recruited ~500,000 participants who were aged 37–73 years, from 22 centers across England, Wales, and Scotland. At recruitment, participants provided a wide arrange of health-related information, underwent various physical measurements, and were collected for biological samples.

Of the 273,324 female participants, we excluded those with a history of cholecystectomy (*n* = 6,813), with disorders of gallbladder, biliary tract, or pancreas (*n* = 7,484), or with cancer at baseline (*n* = 29,496). We further excluded 834 participants who developed incident cancer in the duodenum, liver, gallbladder, biliary tract, or pancreas, and 18 participants who received liver transplantation during follow-up. Of the remaining 228,679 participants, we additionally excluded those without information on menopausal status or age at menopause (*n* = 43,460) or HRT use (*n* = 542). Finally, 184,677 women were included in the current analysis, among whom 126,717 (68.6%) had been menopausal at recruitment and 59,155 (32.0%) had ever used HRT (Supplementary Figure 1).

### Assessments of menopausal characteristics and HRT use

Participants self-reported information on various menopausal characteristics through touchscreen-based questionnaires. These included menopause status (yes or no), age at menopause, histories of hysterectomy and oophorectomy and the corresponding age when received the surgery. Information on history of HRT use and the age at first/last HRT use was also collected via self-report, using the following questions: “Having you ever used hormone replacement therapy (HRT)?”; “How old were you when you first used HRT?”; and “How old were you when you last used HRT?”. According to the self-reported information, duration of using HRT was estimated by subtracting age at starting HRT from age at stopping the use. For this variable, women who reported “Still taking HRT,” “Do not know,” or “Prefer not to answer” were considered as lacking information on age at last use of HRT. For menopausal women, type of menopause (surgical or natural) was determined according to the reported information on age at menopause and age at hysterectomy or oophorectomy. A woman was deemed to be naturally-menopausal if the reported menopausal age was younger than the reported age at hysterectomy or oophorectomy, and otherwise surgical menopause.

### Outcome ascertainment

The outcome of interest in this study was the occurrence of cholecystectomy during the follow-up period. We chose cholecystectomy because it is more clinically relevant than asymptomatic gallstones (17). The procedures of cholecystectomy in the UK Biobank were identified using hospital inpatient records obtained from the Hospital Episode Statistics for England, Scottish Morbidity Record data for Scotland, and the Patient Episode Database for Wales. Specifically, the incident cases of cholecystectomy were identified using the UK Biobank field IDs 41200 (operative procedures-main) and 41260 (date of first operative procedure-main), with the following codes: J18.1, J18.2, J18.3, J18.4, J18.5, J18.8, and J18.9.

### Assessment of covariates

Information on sociodemographic factors, medical history, lifestyle behaviors, and medication use was collected at baseline by touchscreen questionnaires and nurse-led interviews. The Townsend deprivation index was calculated by combining four census variables (unemployment, non-car ownership, non-home ownership, and household overcrowding). BMI was calculated based on measured weight and height (kg/m^2^). Physical activity was assessed at baseline using the self-reported short-form International Physical Activity Questionnaire, and the data were summarized and reported in MET-h per week. To account for variations in alcohol content and drink volumes across beverages, amounts of daily alcohol consumption in grams were estimated from consumption of red wine, white wine, beer, spirit, and fortified wine using previously reported methods (18). Healthy dietary score was calculated as the sum of all the followings (assigning 1 point to each item): (1) Red meat <2 servings/week; (2) Processed meat <1 serving/week; (3) Fresh fruit ≥3 servings/day OR fresh vegetables ≥3 servings/day OR a combination ≥4.5 servings/day; (4) Whole grain ≥3 servings/day; (5) Refined grain <1.5 servings/day; (6) Fish ≥2 servings/week. Diabetes was defined as a self-reported physician's diagnosis or antidiabetic medication use, or an HbA1c level of ≥6.5%. More detailed information on the covariates is presented in Supplementary Table 1.

### Statistical analysis

Baseline participant characteristics were reported for the whole study population and by incident cholecystectomy status. Continuous variables were presented as mean ± standard deviation (SD) and categorical variables as percentage. Missing data for continuous covariates were addressed using sex-specific median values, and categorical covariates were addressed with a missing indicator category.

We used multivariable Cox proportional hazards regression models to estimate hazard ratios (HRs) and 95% confidence intervals (CIs) for the associations of the aforementioned menopausal characteristics and HRT use with risk of cholecystectomy. Person-time of follow-up was calculated as the duration between the date of baseline evaluation and the date of the diagnosis of cholecystectomy, death, loss to follow-up, or the respective censoring dates for England (September 30, 2021), Scotland (July 31, 2021), and Wales (February 28, 2018), whichever occurred first. Three models were constructed to incrementally adjust for potential confounders. Model 1 was adjusted for age (years), ethnic group (White, Asian or Asian British, Black or Black British, mixed ethnicities), and Townsend deprivation index. Model 2 was adjusted for the covariates in model 1 and was further adjusted for smoking status (never, former, current), pack-years of smoking (for current smokers), drinking status (never, former, current), drinking amounts (g/day), total physical activity (MET-h/week), BMI (kg/m^2^), diabetes (yes, no), and use of statins (yes, no), aspirin (yes, no), and non-steroidal anti-inflammatory drugs (NSAIDs; yes, no). Model 3 (full model) was further adjusted for HRT use (ever, never; for menopausal characteristics) or the status and type of menopause (pre-menopause, natural menopause, surgical menopause; for HRT use). A sensitivity analysis was performed by further adjusting for the healthy dietary score. Further sensitivity analyses were performed among women who had been menopausal at baseline, to reexamine the association of menopausal characteristics or HRT use with risk of cholecystectomy.

In addition, we performed a further analysis jointly considering HRT use and the status and type of menopause, with naturally-menopausal women who never used HRT as the reference. Statistical analyses were performed using Stata 15.0 software (StataCorp) and a two-sided *P*-value of <0.05 was considered statistically significant.

## Results

### Participant characteristics

Mean age was 55.6 (± 8.2) years for the included women and the majority (95.5%) of them were ethnically white Europeans. Among the 184,677 women, there were 4,991 incident cases of cholecystectomy over a median of 12.7 years of follow-up. As compared with women who did not have cholecystectomy, those who had cholecystectomy had a higher Townsend deprivation index (lower socioeconomic status), were more likely to be smokers, were less likely to be alcohol drinkers, had lower levels of physical activity and higher levels of BMI, and they were more likely to have diabetes and use aspirin or NSAIDs (Table 1).

**Table 1:** Baseline participant characteristics according to incident cholecystectomy status.

	**Overall (*n* = 184,677)**	**Cholecystectomy**	
		**Yes (*n* = 4,991)**	**No (*n* = 179,686)**
Age, year	55.6 ± 8.2	55.8 ± 8.1	55.6 ± 8.2
**Ethnicity, %**			
White	95.5	96.7	95.5
Asian or Asian British	1.9	1.8	1.9
Black or Black British	1.8	0.9	1.8
Mixed	0.7	0.6	0.7
Townsend deprivation index	−1.4 ± 3.0	−1.1 ± 3.1	−1.4 ± 3.0
**Smoking status, %**			
Never	60.9	57.7	61.0
Former	30.6	32.8	30.5
Current	8.5	9.5	8.5
Pack-years of smoking (current smokers)	24.0 ± 14.0	24.9 ± 13.8	24.0 ± 14.0
**Drinking status, %**			
Never	5.7	6.7	5.7
Former	3.3	4.0	3.3
Current	91.0	89.3	91.0
Drinking amounts, g/day	9.6 ± 12.0	7.4 ± 10.8	9.6 ± 12.0
Physical activity, MET-h/week	42.2 ± 41.0	38.5 ± 40.0	42.3 ± 41.0
BMI, kg/m^2^	26.8 ± 5.0	29.7 ± 5.5	26.7 ± 5.0
Diabetes, %	3.8	4.6	3.8
Statin use, %	9.9	9.8	9.9
Aspirin use, %	8.9	9.8	8.9
Use of NSAIDs, %	17.0	20.1	16.9

BMI, body mass index; MET, metabolic equivalents; NSAIDs, non-steroidal anti-inflammatory drugs.

### Menopausal characteristics and risk of cholecystectomy

After the full adjustment (model 3), menopausal women showed a similar risk of cholecystectomy as compared with non-menopausal women (Table 2). When considering type of menopause, however, women with surgical menopause (HR = 1.48; 95% CI: 1.29–1.71), but not natural menopause (HR = 1.04; 95% CI: 0.94–1.14) had a higher risk of cholecystectomy than non-menopausal women.

**Table 2:** Menopausal characteristics and risk of cholecystectomy in women.

	**Cases/total**	**Model 1, HR (95% CI)**	**Model 2, HR (95% CI)**	**Model 3, HR (95% CI)**
**Menopausal status**				
Non-menopausal (Ref.)	1,477/57,960	1.00 (Ref.)	1.00 (Ref.)	1.00 (Ref.)
Menopausal	3,514/126,717	1.15 (1.05–1.27)	1.13 (1.02–1.24)	1.05 (0.96–1.16)
**Type of menopause**				
Non-menopausal (Ref.)	1,477/57,960	1.00 (Ref.)	1.00 (Ref.)	1.00 (Ref.)
Natural menopause	3,121/117,791	1.12 (1.02–1.23)	1.09 (0.99–1.20)	1.04 (0.94–1.14)
Surgical menopause	393/8,926	1.90 (1.66–2.18)	1.71 (1.49–1.95)	1.48 (1.29–1.71)
**Hysterectomy**				
No (Ref.)	4,451/171,881	1.00 (Ref.)	1.00 (Ref.)	1.00 (Ref.)
Yes	535/12,641	1.67 (1.53–1.84)	1.56 (1.42–1.71)	1.45 (1.32–1.59)
**Age at hysterectomy, year**				
No hysterectomy (Ref.)	4,451/171,881	1.00 (Ref.)	1.00 (Ref.)	1.00 (Ref.)
<45	246/5,367	1.80 (1.58–2.05)	1.62 (1.42–1.84)	1.48 (1.29–1.69)
45–48	99/2,208	1.77 (1.45–2.16)	1.67 (1.37–2.05)	1.54 (1.26–1.88)
49–51	57/1,432	1.58 (1.21–2.06)	1.51 (1.16–1.96)	1.39 (1.07–1.81)
≥52	121/3,445	1.39 (1.16–1.67)	1.35 (1.12–1.62)	1.30 (1.08–1.56)
**Oophorectomy**				
No (Ref.)	4,714/177,987	1.00 (Ref.)	1.00 (Ref.)	1.00 (Ref.)
Yes	227/5,469	1.58 (1.38–1.81)	1.45 (1.27–1.66)	1.31 (1.14–1.50)
**Age at oophorectomy, year**				
No oophorectomy (Ref.)	4,714/177,987	1.00 (Ref.)	1.00 (Ref.)	1.00 (Ref.)
<45	62/1,308	1.80 (1.40–2.32)	1.54 (1.20–1.98)	1.33 (1.03–1.71)
45–48	47/1,015	1.75 (1.31–2.33)	1.60 (1.20–2.14)	1.40 (1.05–1.88)
49–51	39/973	1.53 (1.12–2.10)	1.43 (1.05–1.97)	1.28 (0.93–1.76)
≥52	70/2,035	1.31 (1.03–1.66)	1.26 (0.99–1.60)	1.19 (0.94–1.51)

Model 1: age (year), ethnic group (White, Asian or Asian British, Black or Black British, mixed ethnicities), and Townsend deprivation index.

Model 2: model 1 + smoking status (never, former, current), pack-years of smoking (for current smokers), drinking status (never, former, current), drinking amounts (g/day), total physical activity (MET-h/week), BMI (kg/m^2^), diabetes (yes, no), and use of statins (yes, no), aspirin (yes, no), and non-steroidal anti-inflammatory drugs (yes, no).

Model 3: model 2 + hormone replacement therapy (ever, never).

Both hysterectomy (HR = 1.45; 95% CI: 1.32–1.59) and oophorectomy (HR = 1.31; 95% CI: 1.14–1.50) were associated with a higher risk of cholecystectomy. The associations tended be attenuated with increasing age at hysterectomy or oophorectomy, respectively. In particular, oophorectomy at ≥52 years old was not associated with risk of cholecystectomy (HR = 1.19; 95% CI: 0.94–1.51; Table 2). These associations for hysterectomy or oophorectomy and risk of cholecystectomy were similar when excluding non-menopausal women from the analyses (Supplementary Table 2).

We performed an additional analysis considering type of and age at menopause. Regardless of age at menopause, natural menopause was not associated with risk of cholecystectomy (Table 3). As compared with non-menopausal women, women with surgical menopause at <45 (HR = 1.57; 95% CI: 1.34–1.85) or 45 to 51 years old (HR = 1.42; 95% CI: 1.17–1.74), but not those with the surgery at ≥52 years old (HR = 1.20; 95% CI: 0.85–1.70) had a higher risk of cholecystectomy.

**Table 3:** Status and type of menopause and risk of cholecystectomy in women, according to age at menopause.

**Type and age of menopause**	**Cases/total**	**Model 1, HR (95% CI)**	**Model 2, HR (95% CI)**	**Model 3, HR (95% CI)**
Not menopausal (Ref.)	1,477/57,960	1.00 (Ref.)	1.00 (Ref.)	1.00 (Ref.)
Natural menopause at age <45 years	333/11,122	1.24 (1.08–1.42)	1.17 (1.02–1.34)	1.07 (0.93–1.23)
Natural menopause at age 45–51 years	1,498/56,697	1.10 (1.00–1.22)	1.09 (0.98–1.20)	1.04 (0.94–1.15)
Natural menopause at age ≥52 years	1,290/49,972	1.09 (0.98–1.22)	1.05 (0.95–1.18)	1.01 (0.90–1.13)
Surgical menopause at age <45 years	226/4,653	2.06 (1.75–2.41)	1.80 (1.53–2.11)	1.57 (1.34–1.85)
Surgical menopause at age 45–51 years	132/3,242	1.74 (1.43–2.12)	1.62 (1.34–1.98)	1.42 (1.17–1.74)
Surgical menopause at age ≥52 years	35/1,031	1.47 (1.04–2.08)	1.34 (0.95–1.90)	1.20 (0.85–1.70)

Model 1: age (year), ethnic group (White, Asian or Asian British, Black or Black British, mixed ethnicities), and Townsend deprivation index.

Model 2: model 1 + smoking status (never, former, current), pack-years of smoking (for current smokers), drinking status (never, former, current), drinking amounts (g/day), total physical activity (MET-h/week), BMI (kg/m^2^), diabetes (yes, no), and use of statins (yes, no), aspirin (yes, no), and non-steroidal anti-inflammatory drugs (yes, no).

Model 3: model 2 + hormone replacement therapy (ever, never).

### HRT use and risk of cholecystectomy

After multivariable adjustment, HRT use (ever vs. never) was associated with a higher risk of cholecystectomy (HR = 1.29, 95% CI: 1.21–1.38). This association appeared to be independent of age when initiating HRT, duration of HRT use, and status and type of menopause (Table 4). For example, when compared with women who never used HRT, the highest risk was observed among women who ever used HRT and had surgical menopause (HR = 1.79; 95% CI: 1.58–2.02). These results remained significant after further adjustment of healthy dietary score. These associations for HRT use and risk of cholecystectomy were similar after excluding non-menopausal women from the analyses (Supplementary Table 3).

**Table 4:** Hormone replacement therapy (HRT) and risk of cholecystectomy in women.

	**Cases/total**	**Model 1, HR (95% CI)**	**Model 2, HR (95% CI)**	**Model 3, HR (95% CI)**
**HRT use**				
Never (Ref.)	3,105/125,522	1.00 (Ref.)	1.00 (Ref.)	1.00 (Ref.)
Ever	1,886/59,155	1.34 (1.25–1.43)	1.35 (1.27–1.44)	1.29 (1.21–1.38)
**Age when initiated HRT, years**				
Never use (Ref.)	3,105/125,522	1.00 (Ref.)	1.00 (Ref.)	1.00 (Ref.)
<45	384/10,550	1.49 (1.33–1.66)	1.40 (1.25–1.55)	1.28 (1.15–1.44)
45–48	519/15,161	1.41 (1.28–1.55)	1.42 (1.28–1.56)	1.36 (1.13–1.50)
49–51	464/15,356	1.26 (1.13–1.39)	1.33 (1.20–1.47)	1.29 (1.16–1.43)
≥52	349/13,430	1.09 (0.97–1.22)	1.17 (1.04–1.32)	1.16 (1.03–1.30)
**Duration of HRT use, years**				
Never use (Ref.)	3,105/125,522	1.00 (Ref.)	1.00 (Ref.)	1.00 (Ref.)
≤ 4	454/14,680	1.27 (1.15–1.41)	1.25 (1.13–1.39)	1.23 (1.10–1.36)
5–9	401/13,910	1.19 (1.07–1.33)	1.23 (1.10–1.37)	1.19 (1.07–1.33)
10–14	240/8,286	1.20 (1.04–1.37)	1.29 (1.12–1.47)	1.21 (1.06–1.40)
≥15	91/2,552	1.47 (1.19–1.82)	1.48 (1.20–1.84)	1.33 (1.07–1.66)
**HRT use**				
Never (Ref.)	3,105/125,522	1.00 (Ref.)	1.00 (Ref.)	NA
Ever and pre-menopause	91/2,095	1.73 (1.41–2.14)	1.68 (1.36–2.07)	NA
Ever and natural menopause	1,489/50,290	1.24 (1.15–1.32)	1.26 (1.18–1.36)	NA
Ever & surgical menopause	306/6,770	1.92 (1.70–2.16)	1.79 (1.58–2.02)	NA

Model 1: age (years), ethnic group (White, Asian or Asian British, Black or Black British, mixed ethnicities), and Townsend deprivation index.

Model 2: model 1 + smoking status (never, former, current), pack-years of smoking (for current smokers), drinking status (never, former, current), drinking amounts (g/day), total physical activity (MET-h/week), BMI (kg/m^2^), diabetes (yes, no), and use of statins (yes, no), aspirin (yes, no), and non-steroidal anti-inflammatory drugs (yes, no).

Model 3: model 2 + status and type of menopause (pre-menopause, natural menopause, surgical menopause).

### Joint analysis of menopausal characteristics and HRT use with risk of cholecystectomy

**Figure 1:**
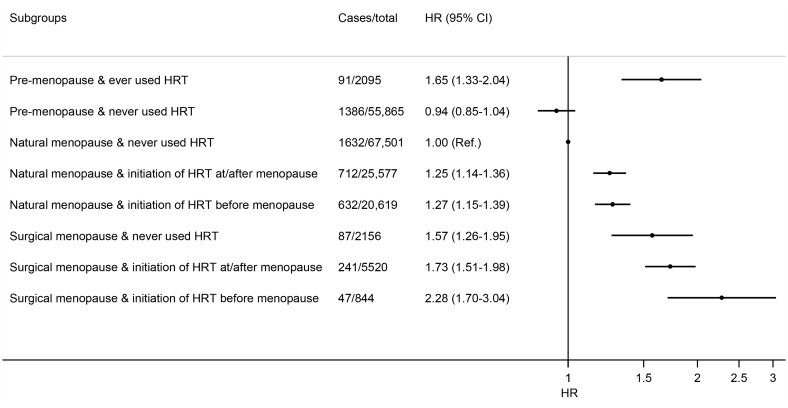
Joint association of menopause status, menopause type, and time at initiating HRT with risk of cholecystectomy in women. Results were adjusted for age (year), ethnic group (White, Asian or Asian British, Black or Black British, mixed ethnicities), Townsend deprivation index, smoking status (never, former, current), pack-years of smoking (for current smokers), drinking status (never, former, current), drinking amounts (g/day), total physical activity (MET-h/week), BMI (kg/m^2^), diabetes (yes, no), and use of statins (yes, no), aspirin (yes, no), and non-steroidal anti-inflammatory drugs (yes, no).

We then performed a further analysis jointly considering menopausal status, type of menopause, history of HRT use, and age at first HRT use. When compared with women who were naturally-menopausal and had never used HRT, non-menopausal women who ever used HRT had a 65% (HR = 1.65; 95% CI: 1.33–2.04) higher risk of cholecystectomy, while naturally-menopausal women who ever used HRT only had a modestly higher risk (regardless of time when starting HRT; Figure 1). The highest risk was observed for women who had surgical menopause and started HRT use before menopause (HR = 2.28, 95% CI: 1.70–3.04), followed by surgically-menopausal women who started HRT during or after the menopausal process (HR = 1.73; 95% CI: 1.51–1.98). These results were largely similar after further adjusting for the healthy dietary score (Supplementary Figure 2).

## Discussion

In this analysis of large, population-based prospective study of middle-aged and older women, we assessed several menopausal characteristics and HRT use in relation to risk of cholecystectomy. Our findings showed that natural menopause, regardless of menopausal age, was not associated with risk of cholecystectomy, while surgical menopause, especially that occurred at a relatively younger age, was associated with a higher risk of cholecystectomy. Ever use of HRT was associated with a higher risk of cholecystectomy. In particular, women who were surgically-menopausal and started HRT before menopause had 2.28-fold risk of cholecystectomy when compared with women who were naturally-menopausal and never used HRT.

There are only a few studies regarding the effect of specific menopausal characteristics on gallbladder disease, and the findings have been conflicting. In the Biliary Tract Cancers pooling Project, increasing reproductive years were associated with an elevated risk of gallbladder cancer in non-Asian women (9), whereas in a case-control study, earlier age of menopause reflecting shorter reproductive lifespan was associated with a higher risk of gallbladder cancer (10). Moreover, in a case-control study in China, menopausal status was not associated with gallstones (19). In the present study, natural menopause, regardless of menopausal age, was not associated with risk of cholecystectomy, while surgical menopause was associated with a higher risk of cholecystectomy. Further stratification by age suggested that only surgical menopause occurred at a relatively younger age was associated with the risk of cholecystectomy. Such an association is not explained by HRT use following surgical menopause at a younger age, as there was a joint influence of HRT use and surgical menopause on risk of cholecystectomy. Furthermore, sensitivity analyses restricted to those who were menopausal at baseline showed that the associations of hysterectomy and bilateral oophorectomy with risk of cholecystectomy remained significant. Several explanations for the association between surgical menopause and risk of cholecystectomy have been proposed. Firstly, surgical menopause could result in a rapid loss of endogenous estrogen compared with natural menopause, which can alter cholesterol metabolism and promote gallstone formation. Secondly, experimental studies have shown that in the rodent ovariectomy model, fat mass, hepatic triglyceride content, and lipidosis were increased compared to controls (20), which may promote the precipitation of cholesterol in bile. Finally, changes in lifestyle factors such as diet and physical activity after undergoing uterine and ovarian removal surgeries may contribute to the development of gallbladder diseases. Future research is needed to explore the underlying mechanisms.

Several prospective observational studies have shown a relatively consistent relationship between HRT use and an elevated risk of gallbladder disease or cholecystectomy (12–15, 21). In the Heart and Estrogen/Progestin Replacement Study, a placebo-controlled trial of postmenopausal hormone therapy for coronary heart disease, treatment with estrogen plus progestin increased the risk for biliary tract surgery by 38% (22). A nested case-control study conducted using the UK Clinical Practice Research Datalink has suggested that combination HRT formulations and oral administrations were associated with higher risk of gallbladder cancer (23). The effect of HRT on the development of gallbladder disease was also evaluated in the Women's Health Initiative (WHI) hormone trials (24). In the WHI trials, 22,579 menopausal women without prior cholecystectomy were randomized to receive conjugated equine estrogen or placebo (for those with hysterectomy), or estrogen plus progestin or placebo (for those without hysterectomy). The results showed that HRT increased risk of any gallbladder disease or surgery after more than 5 years of follow-up (24). In addition, different formulations did not appear to influence the effect of hormone therapy, as the increases in the risk were similar for estrogen alone (HR = 1.67; 95% CI: 1.35–2.06) and estrogen with progestin (HR = 1.59; 95% CI: 1.28–1.97) (24). This is in line with results from the Million Women Study (12) but contradicted those from the French E3N study (13) in which oral estrogen alone, but not oral estrogen with progestin, was associated with a higher risk of cholecystectomy. With respect to hormone administration, the Million Women Study (12) found that the elevation in the risk of cholecystectomy with transdermal was substantially weaker than that with oral therapy. Likewise, in the French E3N study (13), transdermal estrogen, regardless of the formulations, was not associated with risk of cholecystectomy. Information on the formulations or administration (oral or transdermal) of hormones was not available in the UK Biobank, precluding further specific analyses. Moreover, women with hormone therapy in the WHI trials were more likely to receive cholecystectomy rather than other biliary tract surgeries (24), indicating the specificities of the effects of hormone therapy on gallbladder disease. In the current study, we found that ever use of HRT was associated with a higher risk of cholecystectomy, independent of menopausal characteristics, which is in agreement with the WHI findings (24).

Several biologically plausible pathways have been proposed to interpret the associations between HRT use and cholecystectomy. First, exogenous estrogen therapy could modify lipid metabolism, cause biliary cholesterol saturation, and promote precipitation of cholesterol in the bile (25). Second, HRT use inhibits chenodeoxycholic acid secretion and accelerates nucleation of cholesterol monohydrate crystals (8). Third, estrogen therapy could reduce gallbladder motility, which increases bile crystallization and contributes to gallstone formation (26). Finally, estrogen therapy has been suggested to have promoting effects on the inflammation (6), which is a pathological mechanism in the development of gallbladder disease (27).

Strengths of our study lie in its large sample size, the prospective design, and the long-term follow up. Our analysis simultaneously evaluated various menopausal characteristics (including menopausal status and age at and type of menopause) and HRT use as well as their independent and joint influence on the risk of cholecystectomy in women, after taking into account a wide arrange of known risk factors. Moreover, our study is further strengthened by the sensitivity analyses restricted to those who had been menopausal at baseline, which further validate the observed relationships between menopausal characteristics and risk of cholecystectomy.

Several potential limitations should be acknowledged when interpreting our findings. First, given the observational design of the current study, the potential influence residual confounding on our results cannot be entirely excluded. Second, information on menopausal characteristics and HRT use was collected through participants' self-report, which is prone to recall bias leading to misclassification of the exposures. Given the prospective design of the study, such misclassification is likely to be non-differential and could attenuate the examined relationships. Third, as mentioned above, we could not evaluate whether the association for HRT differed by formulations or administration of hormones because of the lack of data in the UK Biobank. Fourth, our analysis considered the risk for cholecystectomy which is more clinically relevant endpoint, and did not assess the risk for global gallbladder disease, although hormone therapy has been similarly associated with both endpoints in clinical trials (24). Finally, our analysis included middle-aged and older women in the UK who are predominantly of white ethnic background, and the findings need to be confirmed for other populations.

## Conclusions

In summary, surgical menopause at a young age, rather than natural menopause, was associated with higher risk of cholecystectomy. Ever use of HRT was associated with a higher risk of cholecystectomy independently of other menopausal characteristics, with the highest risk for surgically-menopausal women who initiated HRT before menopause.

## Data Availability

The datasets presented in this study can be found in online repositories. The names of the repository/repositories and accession number(s) can be found in the article/Supplementary material.
