# Supplementary material for: Menopausal characteristics and hormone replacement therapy in relation to long-term risk of cholecystectomy in women
Source: Front Med (Lausanne). 2024 Nov 27;11:1446271. doi: 10.3389/fmed.2024.1446271 (PMC11631607; doi:10.3389/fmed.2024.1446271)
Supplement: Supplementary file 1 [file Data_Sheet_1.docx]

**Supplementary Figure 1. flow chart of participant selection**

**Supplementary Table 1. Information on exposures, outcomes, and covariates**

| **Variables** | **Field ID** | **OCPS-4 code** |
| --- | --- | --- |
| **Exposures** |  |  |
| Menopause status | 2724 |  |
| Age at menopause | 3581 |  |
| History of hysterectomy | 3591 |  |
| Age at hysterectomy | 2824 |  |
| History of oophorectomy | 2834 |  |
| Age at oophorectomy | 3882 |  |
| History of HRT use | 2814 |  |
| Age when first used HRT | 3536 |  |
| Age when last used HRT | 3546 |  |
| **Outcomes** |  |  |
| Cholecystectomy | 41200 | J18.1, J18.2, J18.3, J18.4, J18.5, J18.8, J18.9 |
| **Potential covariates** |  |  |
| Age | 21003 |  |
| Sex | 31 |  |
| Ethnicity | 21000 |  |
| Townsend deprivation index | 189 |  |
| Smoking status | 20116 |  |
| Pack-years of smoking (current smokers) | 20161 |  |
| Drinking status | 20117 |  |
| Drinking amounts, g/day | 1568, 1578, 1588, 1598, 1608, 5364, 4407, 4418, 4429, 4440, 4451, 4462 |  |
| Healthy dietary score | 1309, 1289, 1299, 1369, 1379, 1389, 1349, 1329, 1339, 1458, 1468, 1438, 1448 |  |
| Physical activity, MET-h/week | 22040 |  |
| BMI | 21001 |  |
| Diabetes | 130706/130708/2443/20002/20003/6153/30750 |  |
| Statin use | 20003 |  |
| Aspirin use | 6154 |  |
| Use of NSAIDs | 6154 |  |

OCPS-4, Office of Population Censuses and Surveys Classification of Interventions and Procedures, version 4; HRT, Hormone replacement therapy; BMI, Body mass index; MET, metabolic equivalents; NSAIDs, non-steroidal anti-inflammatory drugs.

| **Supplementary Table 2. Hysterectomy or oophorectomy and risk of cholecystectomy in menopausal women** | | | | |
| --- | --- | --- | --- | --- |
|  | **Cases/total** | **Model 1,**  **HR (95% CI)** | **Model 2,**  **HR (95% CI)** | **Model 3,**  **HR (95% CI)** |
| **Hysterectomy** |  |  |  |  |
| No (Ref.) | 2986/114,256 | 1.00 (Ref.) | 1.00 (Ref.) | 1.00 (Ref.) |
| Yes | 526/12,370 | 1.68 (1.53-1.85) | 1.57 (1.43-1.72) | 1.47 (1.33-1.62) |
| **Age at hysterectomy, y** |  |  |  |  |
| No hysterectomy (Ref.) | 2986/114,256 | 1.00 (Ref.) | 1.00 (Ref.) | 1.00 (Ref.) |
| <45 | 240/5185 | 1.81 (1.59-2.07) | 1.62 (1.42-1.85) | 1.51 (1.32-1.72) |
| 45 to 48 | 96/2166 | 1.71 (1.40-2.10) | 1.65 (1.35-2.02) | 1.53 (1.25-1.88) |
| 49 to 51 | 57/1416 | 1.58 (1.22-2.06) | 1.53 (1.18-1.99) | 1.42 (1.09-1.85) |
| ≥52 | 121/3436 | 1.37 (1.14-1.64) | 1.36 (1.13-1.64) | 1.32 (1.10-1.59) |
| **Bilateral oophorectomy** |  |  |  |  |
| No (Ref.) | 3243/120,254 | 1.00 (Ref.) | 1.00 (Ref.) | 1.00 (Ref.) |
| Yes | 225/5379 | 1.58 (1.38-1.81) | 1.46 (1.27-1.67) | 1.33 (1.16-1.53) |
| **Age at oophorectomy, y** |  |  |  |  |
| No oophorectomy (Ref.) | 3,243/120,254 | 1.00 (Ref.) | 1.00 (Ref.) | 1.00 (Ref.) |
| <45 | 62/1269 | 1.80 (1.40-2.31) | 1.55 (1.20-1.99) | 1.37 (1.06-1.77) |
| 45 to 48 | 46/1002 | 1.70 (1.27-2.28) | 1.57 (1.17-2.10) | 1.40 (1.05-1.88) |
| 49 to 51 | 39/967 | 1.54 (1.12-2.11) | 1.44 (1.05-1.97) | 1.30 (0.95-1.79) |
| ≥52 | 69/2028 | 1.31 (1.03-1.66) | 1.26 (0.99-1.60) | 1.20 (0.94-1.52) |
| Model 1: age (y), ethnic group (White, Asian or Asian British, Black or Black British, mixed ethnicities), and Townsend deprivation index.  Model 2: model 1 + smoking status (never, former, current), pack-years of smoking (for current smokers), drinking status (never, former, current), drinking amounts (g/d), total physical activity (MET-h/week), BMI (kg/m^2^), diabetes (yes, no), and use of statins (yes, no), aspirin (yes, no), and non-steroidal anti-inflammatory drugs (yes, no).  Model 3: model 2 + hormone replacement therapy (ever, never). | | | | |

| **Supplementary Table 3. Hormone replacement therapy (HRT) and risk of cholecystectomy in menopausal women** | | | | |
| --- | --- | --- | --- | --- |
|  | **Cases/total** | **Model 1,**  **HR (95% CI)** | **Model 2,**  **HR (95% CI)** | **Model 3,**  **HR (95% CI)** |
| **HRT use** |  |  |  |  |
| Never (Ref.) | 1719/69,657 | 1.00 (Ref.) | 1.00 (Ref.) | 1.00 (Ref.) |
| Ever | 1795/57,060 | 1.30 (1.21-1.39) | 1.32 (1.23-1.41) | 1.27 (1.18-1.36) |
| **Age when initiated HRT, y** |  |  |  |  |
| Never use (Ref.) | 1719/69,657 | 1.00 (Ref.) | 1.00 (Ref.) | 1.00 (Ref.) |
| <45 | 364/9846 | 1.48 (1.32-1.66) | 1.41 (1.25-1.58) | 1.29 (1.15-1.45) |
| 45 to 48 | 483/14,568 | 1.35 (1.22-1.49) | 1.36 (1.23-1.51) | 1.30 (1.17-1.44) |
| 49 to 51 | 445/14,916 | 1.23 (1.10-1.36) | 1.30 (1.17-1.44) | 1.26 (1.13-1.40) |
| ≥52 | 340/13,230 | 1.07 (0.95-1.20) | 1.15 (1.02-1.29) | 1.13 (1.01-1.28) |
| **Duration of HRT use, y** |  |  |  |  |
| Never use (Ref.) | 1719/69,657 | 1.00 (Ref.) | 1.00 (Ref.) | 1.00 (Ref.) |
| ≤4 | 435/14,257 | 1.24 (1.12-1.38) | 1.24 (1.11-1.37) | 1.21 (1.09-1.35) |
| 5 to 9 | 395/13,783 | 1.18 (1.05-1.32) | 1.22 (1.09-1.36) | 1.18 (1.05-1.32) |
| 10 to 14 | 235/8210 | 1.18 (1.03-1.36) | 1.27 (1.10-1.46) | 1.19 (1.04-1.38) |
| ≥15 | 90/2519 | 1.48 (1.19-1.83) | 1.49 (1.20-1.85) | 1.33 (1.07-1.66) |
| **HRT use** |  |  |  |  |
| Never (Ref.) | 1719/69,657 | 1.00 (Ref.) | 1.00 (Ref.) | NA |
| Ever & natural menopause | 1489/50,290 | 1.22 (1.14-1.31) | 1.25 (1.17-1.35) | NA |
| Ever & surgical menopause | 306/6770 | 1.89 (1.67-2.14) | 1.78 (1.57-2.01) | NA |
| Model 1: age (y), ethnic group (White, Asian or Asian British, Black or Black British, mixed ethnicities), and Townsend deprivation index.  Model 2: model 1 + smoking status (never, former, current), pack-years of smoking (for current smokers), drinking status (never, former, current), drinking amounts (g/d), total physical activity (MET-h/week), BMI (kg/m^2^), diabetes (yes, no), and use of statins (yes, no), aspirin (yes, no), and non-steroidal anti-inflammatory drugs (yes, no).  Model 3: model 2 + status and type of menopause (pre-menopause, natural menopause, surgical menopause). | | | | |

**Supplementary Figure 2. Joint association of menopause status, menopause type, and time at initiating HRT with risk of cholecystectomy in women.**

Results were adjusted for the covariates listed in the legend for Figure 1 in the article with further adjustment for healthy dietary score defined by a higher intake of fruit, vegetables, whole grains, and fish, and a lower intake of red meat, processed meat, and refined grains.
